# Needling Point Location Used in Sham Acupuncture for Chronic Nonspecific Low Back Pain: A Systematic Review and Network Meta-Analysis

**DOI:** 10.1001/jamanetworkopen.2023.32452

**Published:** 2023-09-06

**Authors:** Boram Lee, Chan-Young Kwon, Hye Won Lee, Arya Nielsen, L. Susan Wieland, Tae-Hun Kim, Stephen Birch, Terje Alraek, Myeong Soo Lee

**Affiliations:** 1 KM Science Research Division, Korea Institute of Oriental Medicine, Daejeon, Republic of Korea; 2 Department of Oriental Neuropsychiatry, Dong-Eui University College of Korean Medicine, Busan, Republic of Korea; 3 KM Convergence Research Division, Korea Institute of Oriental Medicine, Daejeon, Republic of Korea; 4 Department of Family Medicine and Community Health, Icahn School of Medicine at Mount Sinai, New York, New York; 5 Center for Integrative Medicine, University of Maryland School of Medicine, Baltimore, Maryland; 6 Korean Medicine Clinical Trial Center, Korean Medicine Hospital, Kyung Hee University, Seoul, Republic of Korea; 7 Kristiania University College, School of Health Sciences, Oslo, Norway; 8 Department of Community Medicine, Faculty of Health Sciences, National Research Center in Complementary and Alternative Medicine, Institute of Health Sciences, Tromsø, Norway

## Abstract

**Importance:**

When sham acupuncture is set as a control in evaluating acupuncture, the sham needling technique is usually different from acupuncture. However, the sham procedure is conducted either at the same points that are used for the acupuncture group or at nonindicated points.

**Objective:**

To assess whether the outcome of sham acupuncture varies according to the needling points in sham-controlled trials of acupuncture for chronic nonspecific low back pain (CLBP) as an example.

**Data sources:**

Searches of MEDLINE, Embase, Cochrane Central Register of Controlled Trials, and the Allied and Complementary Medicine Database were conducted on February 12, 2023.

**Study selection:**

Randomized clinical trials (RCTs) assessing the outcomes of acupuncture in sham acupuncture–controlled or waiting list–controlled trials on CLBP were included.

**Data extraction and synthesis:**

Two researchers independently extracted data on study characteristics and outcomes and assessed quality. Sham acupuncture was classified according to whether it was conducted at the same acupuncture points used in the acupuncture group, referred to as sham acupuncture therapy (verum) (SATV) or at different points, referred to as sham acupuncture therapy (sham) (SATS). Clinical similarity, transitivity, and consistency tests were conducted, followed by a random-effects frequentist network meta-analysis (NMA).

**Main outcomes and measures:**

The primary outcome was pain, and the secondary outcome was back-specific function. The first assessment after the end of treatment was chosen for analysis. Effect sizes are reported as standardized mean differences (SMD) with 95% CIs. The risk of bias was assessed using the Cochrane risk of bias tool, and the certainty of evidence for findings was evaluated using the Grading of Recommendations Assessment, Development, and Evaluation approach.

**Results:**

Ten RCTs involving 4379 participants were included. In comparison with SATS, acupuncture was significantly associated with improvements in both pain (SMD, −0.33; 95% CI, −0.52 to −0.15) and function outcomes (SMD, −0.13; 95% CI, −0.25 to −0.02); however, there were no differences between acupuncture and SATV. In comparison with SATS, SATV was significantly associated with better pain (SMD, −0.45; 95% CI, −0.88 to −0.03) and function outcomes (SMD, −0.30; 95% CI, −0.56 to −0.05). The risk of bias that could affect the interpretation of the results was usually low, and the certainty of evidence was moderate to low.

**Conclusions and relevance:**

In this NMA, sham acupuncture needling at the same points as those in acupuncture was not a true placebo control for assessing the efficacy of acupuncture for CLBP and might underestimate the outcome of acupuncture in clinical settings.

## Key Points

**Question:**

Is the location of the needling point in sham-controlled trials of acupuncture for chronic nonspecific low back pain (CLBP) associated with outcomes?

**Findings:**

In a network meta-analysis of 10 randomized clinical trials with 4379 participants, sham acupuncture needling at the same acupuncture points as those in the acupuncture group was significantly associated with better pain and function outcomes compared with sham acupuncture needling at different points.

**Meaning:**

These findings suggest sham acupuncture needling at the same points as those in the acupuncture group may not provide a true placebo control for assessing the efficacy of acupuncture for CLBP.

## Teaser

This systematic review and network meta-analysis evaluates whether the location of needling point in sham-controlled trials of acupuncture for chronic nonspecific low back pain is associated with outcomes.

## Introduction

Chronic nonspecific low back pain (CLBP) is defined as pain between the lower rib and the inferior gluteal fold lasting more than 3 months without a specific pathology or cause.^1^ The prevalence of CLBP was 15.4% to 37.3% in the general population.^1,2,3^ Conventional treatments for CLBP incorporate pharmacotherapy and nonpharmacologic therapy, including physical therapy.^4^ However, not all patients respond to conventional treatments, and adverse effects from the long-term use of pharmacotherapy have been reported.^5,6,7^

Although many randomized clinical trials (RCTs) evaluating the outcomes of acupuncture on CLBP have been performed, the results continue to be controversial, and questions about the appropriateness of sham acupuncture control have been raised.^8^ Accordingly, clinical practice guidelines for CLBP have promoted inconsistent recommendations on acupuncture due to a lack of what has been deemed high-quality evidence.^4,5,6,9,10,11^

In RCTs evaluating the efficacy of acupuncture, it is very important to set up a physiologically inert control that has the potential to serve as a true placebo.^12^ However, in almost all RCTs, when researchers set up sham acupuncture as a control, they never demonstrate the sham needling technique as being physiologically inert, although it is usually different from acupuncture.^13,14,15^ Although the evidence from brain imaging and biological studies has indicated point specificity in acupuncture,^16,17^ the sham procedure is sometimes conducted at the same points used for acupuncture.^18^ In this case, even though sham acupuncture produces less stimulation with superficial needling or a sham acupuncture device, an effect due to acupuncture point specificity cannot be ruled out.^13,18^ Therefore, the use of these noninert sham acupuncture controls can risk underestimating the outcome of acupuncture. The purpose of this systematic review of sham acupuncture–controlled trials of acupuncture for CLBP is to determine whether sham acupuncture produces different results depending on whether it is conducted at the same acupuncture points as those in the acupuncture group or at other points.

## Methods

This network meta-analysis (NMA) was reported in accordance with the relevant extension of the Preferred Reporting Items for Systematic Reviews and Meta-analyses (PRISMA) reporting guideline.^19^ Study populations were adult participants with CLBP defined as low back pain lasting more than 3 months without a specific cause, without limitations on age, sex, race, and nationality. Studies specifying that the participants were patients with CLBP without a specific pathology and cause were included even if they did not use the term CLBP.

All trials comparing manual acupuncture with sham acupuncture, waiting list, or both were included. We required sham acupuncture interventions to use different needling techniques from acupuncture groups, such as superficial needling at points or using sham devices, including the Park^20^ or Streitberger device.^21^ Sham acupuncture was classified into the following 2 types: (1) sham acupuncture needling at the same acupuncture points as those in the acupuncture group, referred to as sham acupuncture therapy (verum) (SATV) and (2) sham acupuncture needling at points different from those in the acupuncture group, referred to as sham acupuncture therapy (sham) (SATS). The waiting list group (ie, medical management, such as rescue medication use, that was judged not to have a significant effect on the results) was set as the reference group to form a connected loop in NMA. Studies comparing acupuncture with sham acupuncture in addition to standard treatments such as therapeutic exercise in all groups were also included. For acupuncture, studies in which needles were not used, or studies involving nonmanual needle stimuli, including electroacupuncture, were excluded.

The primary outcome was pain as assessed by the Visual Analog Scale (VAS) or other validated scales. The secondary outcome was back-specific function assessed by the Roland Morris Disability Questionnaire (RMDQ) or other validated scales. The time point for analysis was the earliest result after the completion of all planned treatment sessions.

### Information Sources and Search

Four electronic databases, including MEDLINE, Embase, the Cochrane Central Register of Controlled Trials, and the Allied and Complementary Medicine Database, were searched from their inception dates to February 12, 2023. The reference lists of eligible studies and review articles were also searched to find eligible studies. Both studies published in peer-reviewed journals and studies from the gray literature, including conference proceedings, were included. The search strategy used in each database is included in eMethods 1 in Supplement 1.

### Study Selection and Data Extraction

Citations retrieved from databases were imported into EndNote version 20 (Clarivate Analytics), and duplicate citations were removed. Two authors (B.L. and C.Y.K.) independently reviewed the titles and abstracts of each record. The full text was retrieved for potentially eligible studies, and the same 2 authors independently reviewed the full texts, selected studies that met the eligibility criteria for inclusion, and conducted data extraction. Any disagreements were resolved by discussion or by consultation with other authors.

For included studies, basic study information, details of the population, intervention, outcomes of interest, and results were extracted using a pilot–tested Excel form. If the data were ambiguous, the authors of individual studies were contacted by email.

### Risk of Bias Within Individual Studies

The risk of bias within individual studies was assessed by 2 independent authors (B.L. and C.Y.K.) using the Cochrane risk of bias tool.^22^ The tool assesses domains of random sequence generation, allocation concealment, blinding of participants, personnel, outcome assessor, incomplete outcome data, selective reporting, and other bias as low, unclear, or high risk for each study.

### Statistical Analysis

The main characteristics of the included studies were qualitatively summarized. For direct evidence, pairwise meta-analysis was conducted using Review Manager version 5.4 (Cochrane). For indirect or mixed evidence, a frequentist NMA was conducted using the network package in Stata/MP version 16 (StataCorp). Clinical similarity, transitivity, and statistical consistency were determined, and NMA estimates were calculated. Specifically, statistical consistency was tested through the node-splitting (local approach) and design-by-treatment interaction model (global approach). The geometry of the NMA was represented using a 4-node network map (acupuncture, SATV, SATS, and waiting list) on each outcome. In the network map, the size of the node and the thickness of the line indicate the number of participants in the intervention and the number of direct comparison trials. For both pairwise meta-analysis and NMA, a random-effects model that estimates the average of the distribution of effects was selected due to expected clinical heterogeneity between the studies. The effect estimates were presented using standardized mean differences (SMDs) with 95% CIs. If sufficient studies (10 or more) were included in an analysis, publication bias was assessed using a funnel plot and Egger test for asymmetry. The surface under the cumulative ranking curve (SUCRA) statistic was examined to identify the best treatment. Tests were 2-sided, and *P* values less than .05 were considered significant.

The certainty of evidence on effect estimates was assessed using the Grading of Recommendations Assessment, Development and Evaluation (GRADE) approach.^23,24^ First, the risk of bias, inconsistency, indirectness, and publication bias of the direct estimate was assessed. Afterwards, the certainty of evidence for the indirect estimate was evaluated considering the lowest of the ratings of the 2 direct comparisons forming the most dominant first-order loop and intransitivity. The certainty of evidence for NMA estimates was assessed by choosing the highest between direct and indirect ratings and examining the incoherence and imprecision. The certainty of evidence was judged as high, moderate, low, or very low on each comparison of outcomes.

## Results

### Study Selection and Characteristics

**Figure 1.**
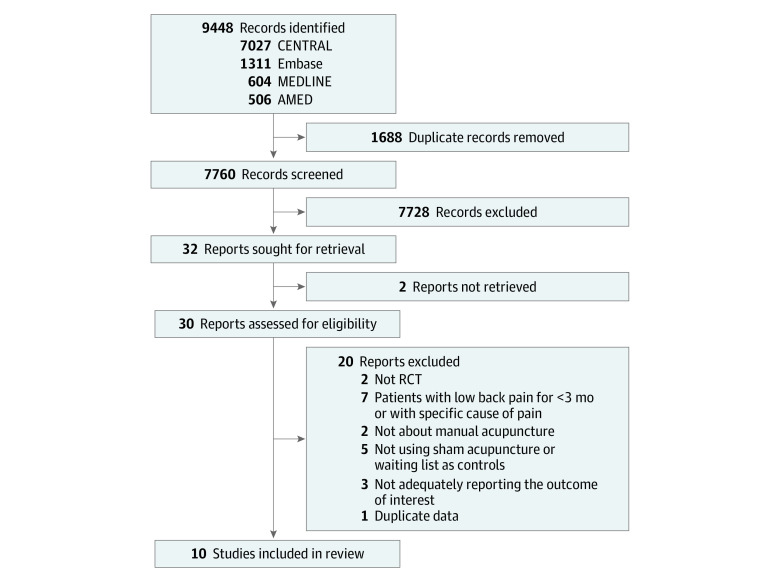
Flow Diagram of the Literature Screening and Selection Processes AMED indicates Allied and Complementary Medicine Database; CENTRAL, the Cochrane Central Register of Controlled Trials; RCT, randomized clinical trial.

A total of 9448 records were identified, and 10 studies involving 4379 participants were included in this review (Figure 1).^25,26,27,28,29,30,31,32,33,34^ eMethods 2 in Supplement 1 shows the bibliographic information of records and the reasons for their exclusion during the full-text review. We contacted the authors of some studies^27,31,33^ via email to clarify any information not adequately reported; however, only 2 authors^27,33^ responded to our requests, and we used the data as reported in the Cochrane review^35^ for the study without a response.^31^

**Figure 2.**
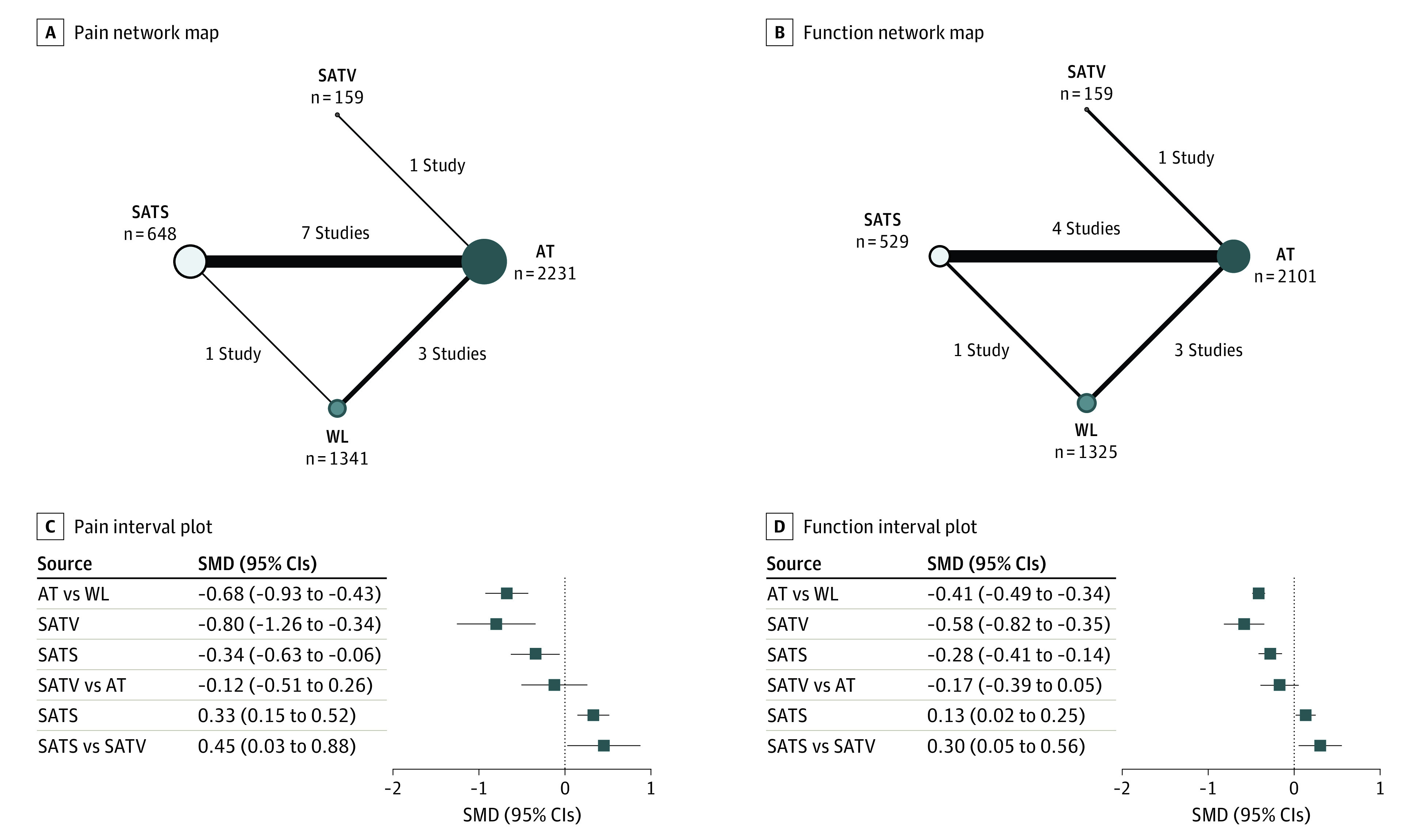
Network Maps and Interval Plots AT indicates acupuncture therapy; SATS, sham acupuncture needling at different points compared with the acupuncture group; SATV, sham acupuncture needling at the same acupuncture points as the acupuncture group; SMD, standardized mean difference; WL, waiting list.

Within studies, CLBP was defined as pain lasting a minimum of either 3 months^26,27,30,32^ or 6 months.^25,28,29,31,33,34^ Seven trials compared acupuncture and sham acupuncture,^26,27,28,30,31,32,34^ and 2 trials compared acupuncture and waiting list.^29,33^ There was one 3-group trial comparing acupuncture, sham acupuncture, and waiting list.^25^ For the needling points of sham acupuncture, 7 studies performed SATS by needling at nonacupuncture points,^25,27,28,30,31,32,34^ and 1 study performed SATV by needling at the same acupuncture points used in the acupuncture group.^26^ For the needling technique of sham acupuncture, 5 studies involved the use of superficial needling,^25,28,30,31,32^ and 3 studies involved sham devices, including Park^27^ or Streitberger devices,^34^ or a toothpick in a needle guide tube.^26^ Pain was assessed using the VAS,^25,27,29,30,31,32,34^ Von Korff Chronic Pain Grade Scale,^28^ numeric rating scale,^26^ or Low Back Pain Rating Scale.^33^ Back-specific function was assessed using the RMDQ,^26,29,30^ Hanover Functional Ability Questionnaire,^25,28,33^ or Oswestry Disability Index (ODI)^27^ (Table and eTable 1 in Supplement 1). There were no statistical inconsistencies according to the global (pain, χ^2^_2_ = 1.33; *P* = .51; function, χ^2^_2_ = 0.32; *P* = .85) and local approaches (eTable 2 in Supplement 1). Figure 2 shows the network geometry of pain and function.

**Table.** Characteristics of Included Studies

Source (country)	Mean (SD) age, y	AT (sample size)	Sham AT (sample size)	Waiting list (sample size)	Treatment duration	Outcomes of interest	Time point included in the analysis
Brinkhaus,^25^ 2006 (Germany)	58.8 (9.1)	AT (140)	SATS: superficial needling at nonacupuncture points (70)	Waiting list (74)	8 wks	Pain (0-100 mm VAS), function (HFAQ)	8 wks
Cherkin,^26^ 2009 (USA)^a^	47 (13)	AT (152)	SATV: a toothpick in a needle guide tube at same acupuncture points used in the AT group (159)	NA	7 wks	Pain (0-10 NRS), function (RMDQ)	8 wks
Cho,^27^ 2013 (South Korea)	42.06 (14.04)	AT (57)	SATS: Park Sham needle at nonacupuncture points (59)	NA	6 wks	Pain (0-10 cm VAS), function (ODI)	6 wks
Haake,^28^ 2007 (Germany)	50 (15)	AT (370)	SATS: superficial needling at nonacupuncture points (375)	NA	5 wks	Pain (Von Korff CPGS), function (HFAQ)	6 wks
Itoh,^29^ 2009 (Japan)	range 61-81	AT (7)	NA	Waiting list (7)	5 wks	Pain (0-100 mm VAS), function (RMDQ)	5 wks
Kwon,^30^ 2007 (South Korea)	Not reported	AT (25)	SATS: superficial needling at nonacupuncture points (25)	NA	4 wks	Pain (0-100 mm VAS), function (RMDQ)	4 wks
Leibing,^31^ 2002 (Germany)	48.1 (9.7)	AT (40)	SATS: superficial needling at nonacupuncture points (45)	NA	12 wks	Pain (0-10 cm VAS)	12 wks
Molsberger,^32^ 2002 (Germany)	50 (7)	AT (65)	SATS: superficial needling at nonacupuncture points (61)	NA	4 wks	Pain (0-100 mm VAS)	4 wks
Witt,^33^ 2006 (Germany)	52.9 (13.7)	AT (pain: 1363, function: 1350)	NA	Waiting list (pain: 1260, function: 1244)	3 mos	Pain (Low Back Pain Rating Scale), function (HFAQ)	3 mos
Yu,^34^ 2020 (USA)	AT: 34.98 (13.16)/Sham AT: 39.51 (14.40)	AT (12)	SATS: Streitberger sham acupuncture at nonacupuncture points (13)	NA	4 wks	Pain (0-10 cm VAS)	4 wks

Abbreviations: AT, acupuncture therapy; CPGS, chronic pain grade scale; HFAQ, Hanover functional ability questionnaire; NA, not applicable; NRS, numeric rating scale; ODI, Oswestry disability index; RMDQ, Roland-Morris disability questionnaire; SATS, sham acupuncture needling at different points compared with the acupuncture group; SATV, sham acupuncture needling at the same acupuncture points as the acupuncture group; VAS, visual analog scale.

^a^ In the original study, the AT group was divided into (semi)standardized and individualized AT groups. However, only data corresponding to the (semi)standardized AT group that meets the question of this study were extracted.

### Risk of Bias Within Studies

Appropriate random sequence generation methods were reported in all of the included studies. Three studies^29,30,31^ lacked information on whether allocation was concealed; thus, we assessed them as having an unclear risk of bias. Because 3 studies had waiting list groups,^25,29,33^ blinding of participants was not possible. Moreover, the trial results were obtained from patient-reported questionnaires; thus, we rated them as having a high risk of performance and detection bias. Blinding of acupuncture therapists was not possible in all studies; however, it was judged as having no effect on the results. Three studies^27,29,34^ that did not perform intent-to-treat analysis were evaluated as having a high risk of attrition bias. All studies were evaluated as having a low risk of reporting bias, and the 2 studies that showed a significant difference in baseline ODI^27^ or analgesic use^25^ between the 2 groups were evaluated as having a high risk of other bias (eFigure 1 in Supplement 1).

### Effect Estimates

#### Pain

According to NMA results, compared with waiting list, acupuncture (SMD, −0.68; 95% CI, −0.93 to −0.43), SATV (SMD, −0.80; 95% CI, −1.26 to −0.34), and SATS (SMD, −0.34; 95% CI, −0.63 to −0.06) were significantly associated with pain improvement. Although there was no difference between SATV and acupuncture, there was a significant difference between SATS and acupuncture, with the results being more favorable for acupuncture (SMD, 0.33; 95% CI, 0.15 to 0.52). In addition, compared with SATV, SATS was significantly associated with worse outcomes (SMD, 0.45; 95% CI, 0.03 to 0.88) (Figure 2C). These results were consistent with the pairwise meta-analysis results in terms of statistical significance (eTable 3 in Supplement 1). The results were consistent with the effect estimates calculated using the fixed-effects model (eTable 4 in Supplement 1). The funnel plot and Egger test for regression gave a *P* value of .63, indicating no evidence of publication bias (eFigure 2 in Supplement 1). The SUCRA plot showed that SATV was ranked first (90.4%), followed by acupuncture (75.6%), SATS (33.6%), and waiting list (0.3%) (eFigure 3 in Supplement 1). In the sensitivity analysis, the exclusion of a small pilot study^29^ did not significantly affect the findings (eFigure 4 in Supplement 1).

#### Function

According to NMA results, compared with waiting list, acupuncture (SMD, −0.41; 95% CI, −0.49 to −0.34), SATV (SMD, −0.58; 95% CI, −0.82 to −0.35), and SATS (SMD, −0.28; 95% CI, −0.41 to −0.14) were associated with better function. There was no difference between SATV and acupuncture. However, there was a significant difference between SATS and acupuncture (SMD, 0.13; 95% CI, 0.02 to 0.25). Furthermore, there was a significant difference between SATS and SATV, with favorable results for SATV (SMD, 0.30; 95% CI, 0.05 to 0.56) (Figure 2D). In the pairwise meta-analysis, the difference between the waiting list and SATS was not significant. The results were identical with the effect estimates calculated using the fixed-effects model (eTable 5 in Supplement 1). According to the SUCRA plot, SATV was ranked first (97.5%), followed by acupuncture (68.5%), SATS (34%), and waiting list (0%) (eFigure 3 in Supplement 1). In the sensitivity analysis, the exclusion of a small pilot study^29^ did not significantly affect the findings (eFigure 4 in Supplement 1).

### Certainty of Evidence

The certainty of direct and indirect evidence on pain and function outcomes was moderate and downgraded due to the risk of bias in individual studies. The certainty of evidence for NMA estimates was moderate or low for both pain and function outcomes, and the reason for further downgrading was imprecision of the NMA effect estimates (eTable 6 and eTable 7 in Supplement 1).

## Discussion

To evaluate the specific effects of acupuncture, RCT researchers use sham acupuncture comparisons using superficial needling or a sham acupuncture device. Although its technique differs from that of acupuncture, sham acupuncture is sometimes carried out at the same points on the body that are used for acupuncture. It is possible that sham contact with acupuncture points is a modified form of acupuncture instead of a true placebo. To understand whether the effects of sham acupuncture at verum acupuncture points are specific, we investigated the outcome of sham acupuncture according to the needling points using CLBP as an example. Although several high-quality systematic reviews on acupuncture in CLBP have been conducted,^10,35,36^ we are not aware of any study comparing the outcome of sham acupuncture according to the needling points.

According to the results of NMA, there was no significant difference between acupuncture and SATV in both pain and function; however, there was a significant difference between acupuncture and SATS. Interestingly, compared with SATS, SATV was associated with better outcomes. The certainty of evidence for these estimates was moderate to low. In SUCRA plots, SATV was ranked first in pain and function improvement, followed by acupuncture. These results indicated that the clinical outcome of sham acupuncture could differ depending on whether the needling point of sham acupuncture is the same as that used in the acupuncture group, suggesting that SATV may not be physiologically inert. Even though the needling technique of sham acupuncture is different from that of acupuncture, needling at the same acupuncture points as those used in the acupuncture group cannot be regarded as providing a true or valid placebo control. The resulting underestimation of acupuncture efficacy may lead to inconsistent recommendations on acupuncture for CLBP in clinical practice guidelines.^4,5,6,9,10,11^

In the NMA, acupuncture, SATV, and SATS significantly improved both pain and function compared with the waiting list. These results are consistent with other studies that find it is almost impossible to remove the placebo effect of acupuncture through a sham acupuncture control design because there are too many factors related to the physiological activity of acupuncture, including needle insertion, psychological factors, acupuncture points, and acupuncture manipulation.^8,13,37^ However, our results should be interpreted carefully because blinding of participants was not possible between the acupuncture, SATV, and SATS groups and the waiting list, and therefore the results were susceptible to nonspecific effects. In addition, in the pairwise meta-analysis, there was no significant difference in function between the SATS and waiting list.

In previous sham acupuncture-controlled trials, results from previous NMAs indicate that there may be a difference in the outcome of acupuncture depending on whether a sham acupuncture device is used.^38,39^ In studies using such a sham acupuncture device, the base unit must be used in both the sham and acupuncture groups for successful blinding of participants.^40^ Because the base unit interferes with the usual manipulations in the acupuncture group,^40^ the effectiveness of acupuncture may be attenuated and fail to represent the effectiveness of acupuncture in the clinical setting. In the studies included in our review, various sham acupuncture devices were used in both sham acupuncture groups, and the use of a sham acupuncture device may also have influenced outcomes of acupuncture and sham acupuncture for CLBP.

This study is meaningful in that it is the first NMA designed to examine the point specificity of acupuncture by analyzing the outcome of sham acupuncture according to the needling point. To gain higher confidence relative to the mixed and indirect evidence confirmed in our study, it will be necessary in the future to conduct a 3-group direct comparative trial of acupuncture, SATV, and SATS. However, given the increasingly recognized problems of sham acupuncture, additional NMAs to confirm whether similar results are shown for other pain and nonpain conditions may be a preferable approach. In addition, sham acupuncture appears to be somewhat active, whether it is at verum or sham points, and the clinical significance of the difference between sham and acupuncture is often contested. Therefore, comparisons between different shams and acupuncture through laboratory studies and reviews can be useful in clarifying the mechanisms of acupuncture.

### Limitations

This study has several limitations. First, only English-language databases were searched; although we tried to collect the relevant evidence as comprehensively as possible, there may be eligible studies not indexed in English language databases. Second, only 1 study used SATV; thus, this may have affected the power and precision of the results. Third, analysis of other potential effect modifiers, such as a sham acupuncture device, was not performed because it could affect the precision of the results by increasing the number of nodes in the network map compared with the number of studies included. Fourth, the waiting list group was included to form a connected loop for NMA; however, it might not be an appropriate group for evaluating self-reported pain and function scores. Additionally, although the effect of a small pilot study was not observed in sensitivity analysis, it is still possible for small studies to affect the reliability of the results.

## Conclusions

The findings of this study suggest sham acupuncture needling at the same points as those in acupuncture may not be a true placebo control for assessing the outcome of acupuncture for CLBP. As a result, it may underestimate the outcome of acupuncture in actual clinical settings.

## Notes

Supplement 1. **eMethods 1. **Search Strategy Used in Each Database **eMethods 2. **Excluded Studies After Full-Text Review **eTable 1. **Details of Acupuncture Treatment Method and Funding Source **eTable 2. **Results of Testing Inconsistency at the Local Level Through the Node Splitting Method **eTable 3. **League Table for Pairwise Meta-Analysis (Right Upper Part) and Network Meta-Analysis (Left Lower Part) Estimates: Using the Random-Effects Model: Pain **eTable 4. **League Table for Pairwise Meta-Analysis (Right Upper Part) and Network Meta-Analysis (Left Lower Part) Estimates: Using the Fixed-Effects Model: Pain **eTable 5. **League Table for Pairwise Meta-Analysis (Right Upper Part) and Network Meta-Analysis (Left Lower Part) Estimates: Using the Random-Effects and Fixed-Effects Models: Function **eTable 6. **The Quality of Evidence for the Main Findings: Pain **eTable 7. **The Quality of Evidence for the Main Findings: Function **eFigure 1. **Risk of Bias Summary for All Included Studies **eFigure 2. **Funnel Plot: Pain **eFigure 3. **SUCRA Plots **eFigure 4. **Interval Plots After Sensitivity Analysis Excluding a Pilot Study

Supplement 2.
Data Sharing Statement
