# Supplementary material for: Needling Point Location Used in Sham Acupuncture for Chronic Nonspecific Low Back Pain: A Systematic Review and Network Meta-Analysis
Source: JAMA Netw Open. 2023 Sep 6;6(9):e2332452. doi: 10.1001/jamanetworkopen.2023.32452 (PMC10483312; doi:10.1001/jamanetworkopen.2023.32452)
Supplement: Supplement 1. — eMethods 1. Search Strategy Used in Each Database eMethods 2. Excluded Studies After Full-Text Review eTable 1. Details of Acupuncture Treatment Method and Funding Source eTable 2. Results of Testing Inconsistency at the Local Level Through the Node Splitting Method eTable 3. League Table for Pairwise Meta-Analysis (Right Upper Part) and Network Meta-Analysis (Left Lower Part) Estimates: Using the Random-Effects Model: Pain eTable 4. League Table for Pairwise Meta-Analysis (Right Upper Part) and Network Meta-Analysis (Left Lower Part) Estimates: Using the Fixed-Effects Model: Pain eTable 5. League Table for Pairwise Meta-Analysis (Right Upper Part) and Network Meta-Analysis (Left Lower Part) Estimates: Using the Random-Effects and Fixed-Effects Models: Function eTable 6. The Quality of Evidence for the Main Findings: Pain eTable 7. The Quality of Evidence for the Main Findings: Function eFigure 1. Risk of Bias Summary for All Included Studies eFigure 2. Funnel Plot: Pain eFigure 3. SUCRA Plots eFigure 4. Interval Plots After Sensitivity Analysis Excluding a Pilot Study [file jamanetwopen-e2332452-s001.pdf]

Supplemental Online Content

Lee B, Kwon C-Y, Lee HW, et al. Needling point location used in sham acupuncture for chronic nonspecific low back pain: a systematic review and network meta-analysis. *JAMA Netw Open*. 2023;6(9):e2332452. doi:10.1001/jamanetworkopen.2023.32452

eMethods 1. Search Strategy Used in Each Database

eMethods 2. Excluded Studies After Full-Text Review

eTable 1. Details of Acupuncture Treatment Method and Funding Source

eTable 2. Results of Testing Inconsistency at the Local Level Through the Node Splitting Method

eTable 3. League Table for Pairwise Meta-Analysis (Right Upper Part) and Network Meta-Analysis (Left Lower Part) Estimates: Using the Random-Effects Model: Pain

eTable 4. League Table for Pairwise Meta-Analysis (Right Upper Part) and Network Meta-Analysis (Left Lower Part) Estimates: Using the Fixed-Effects Model: Pain

eTable 5. League Table for Pairwise Meta-Analysis (Right Upper Part) and Network Meta-Analysis (Left Lower Part) Estimates: Using the Random-Effects and Fixed-Effects Models: Function

eTable 6. The Quality of Evidence for the Main Findings: Pain

eTable 7. The Quality of Evidence for the Main Findings: Function

eFigure 1. Risk of Bias Summary for All Included Studies

eFigure 2. Funnel Plot: Pain

eFigure 3. SUCRA Plots

eFigure 4. Interval Plots After Sensitivity Analysis Excluding a Pilot Study

This supplemental material has been provided by the authors to give readers additional information about their work.

© 2023 Lee B et al. *JAMA Network Open.*

eMethods 1. Search Strategy Used in Each Database

MEDLINE via PubMed

	Searches	Results
#1	"Back Pain"[MH] OR "Low Back Pain"[MH] OR Sciatica[MH] OR "back pain"[TIAB] OR dorsalgia[TIAB] OR backache[TIAB] OR sciatica[TIAB] OR lumbago[TIAB] OR coccydynia[TIAB] OR (lumb* adj3 pain)[TIAB]	79,275
#2	Acupuncture[MH] OR "Acupuncture Therapy"[MH] OR "Acupuncture Points"[MH] OR acupunct*[TIAB] OR acupoint*[TIAB] OR "Dry Needling"[MH] OR "dry needling"[TIAB] OR "filiform needle"[TIAB]	37,635
#3	"Randomized Controlled Trial"[PT] OR "Controlled Clinical Trial"[PT] OR randomized[TIAB] OR placebo[TIAB] OR "Clinical Trials as Topic"[Mesh: noexp] OR randomly[TIAB] OR trial[TI]	1,597,785
#4	animals[MH] NOT humans[MH]	5,090,498
#5	(#1 AND #2 AND #3) NOT #4	**604**

Embase via Elsevier.com

	Searches	Results
#1	'back pain':ab,ti OR 'low back pain'/exp OR 'low back pain':ab,ti OR sciatica/exp OR sciatica:ab,ti OR dorsalgia:ab,ti OR backache/exp OR backache:ab,ti OR lumbago:ab,ti OR coccydynia:ab,ti OR ischialgia/exp OR ischialgia:ab,ti OR (lumb* adj3 pain):ab,ti	155,439
#2	acupuncture/exp OR acupuncture*:ab,ti OR 'acupuncture point'/exp OR 'body meridian'/exp OR 'body meridian':ab,ti OR acupoint*:ab,ti OR 'dry needling'/exp OR 'dry needling':ab,ti OR 'filiform needle':ab,ti	61,319
#3	'crossover procedure':de OR 'double-blind procedure':de OR 'randomized controlled trial':de OR 'single-blind procedure':de OR (random* OR factorial* OR crossover* OR cross NEXT/1 over* OR placebo* OR doubl* NEAR/1 blind* OR singl* NEAR/1 blind* OR assign* OR allocat* OR volunteer*):de,ab,ti	3,080,908
#4	#1 AND #2 AND #3	**1,311**

CENTRAL

	Searches	Results
#1	MeSH descriptor: [Back Pain] explode all trees	6,568
#2	MeSH descriptor: [Low Back Pain] explode all trees	5,205
#3	MeSH descriptor: [Sciatica] explode all trees	368
#4	("back pain" OR dorsalgia OR backache OR sciatica OR lumbago OR coccydynia OR	230,909

© 2023 Lee B et al. *JAMA Network Open.*

	(lumb* NEAR3 pain))ti,ab,kw	
#5	#1 OR #2 OR #3 OR #4	230,914
#6	MeSH descriptor: [Acupuncture] explode all trees	199
#7	MeSH descriptor: [Acupuncture Therapy] explode all trees	6,100
#8	MeSH descriptor: [Acupuncture Points] explode all trees	2,459
#9	MeSH descriptor: [Dry Needling] explode all trees	127
#10	(acupunct* OR acupoint* OR “dry needling” OR "filiform needle"):ti,ab,kw	20,337
#11	#6 OR #7 OR #8 OR #9 OR #10	20,628
#12	(#5 AND #11) in Trials	**7,027**

AMED via EBSCO

	Searches	Results
#1	Back Pain[SU] OR Low Back Pain[SU] OR Sciatica[SU] OR back pain[TX] OR dorsalgia[TX] OR backache[TX] OR sciatica[TX] OR lumbago[TX] OR coccydynia[TX] OR (lumb* N3 pain)[TX]	8,575
#2	Acupuncture[SU] OR Acupuncture Therapy[SU] OR Acupuncture Points[SU] OR acupunct*[TX] OR acupoint*[TX] OR Dry Needling[SU] OR dry needling[TX] OR filiform needle[TX]	12,399
#3	#1 AND #2	**506**

© 2023 Lee B et al. *JAMA Network Open.*

eMethods 2. Excluded Studies After Full-Text Review

- Not randomized controlled trials: 2

1. Ahn JL, Lee IS, Cha SH, Kim GT. The Clinical Effect on Low Back Pain by Acupuncture Treatment and GCM(General Coordinative Manipulation) Treatment. The journal of oriental rehabilitation medicine = hanbang jaehwal euihakgwa hakhoe chi. 2002;12(1):111-20.
2. Clauw DJ, Harris RE. Is acupuncture more effective than sham acupuncture in relieving pain in patients with low back pain? Nature clinical practice Rheumatology. 2006;2(7):362-3.

- Patients with low back pain for less than 3 months or with a specific etiology causing pain: 7

1. Cherkin DC, Eisenberg D, Sherman KJ, Barlow W, Kaptchuk TJ, Street J, et al. Randomized trial comparing traditional Chinese medical acupuncture, therapeutic massage, and self-care education for chronic low back pain. Archives of Internal Medicine. 2001;161(8):1081-8.
2. Coan RM, Wong G, Liang Ku S, Chan YC, Wang L, Ozer FT. The acupuncture treatment of low back pain: A randomized controlled study. American Journal of Chinese Medicine. 1980;8(1-2):181-9.
3. Inoue M, Kitakoji H, Ishizaki N, Tawa M, Yano T, Katsumi Y, et al. Relief of low back pain immediately after acupuncture treatment - A randomised, placebo controlled trial. Acupuncture in Medicine. 2006;24(3):103-8.
4. Itoh K, Katsumi Y, Hirota S, Kitakoji H. Effects of trigger point acupuncture on chronic low back pain in elderly patients - a sham-controlled randomised trial. Acupuncture in Medicine. 2006;24(1):5-12.
5. Makary MM, Lee J, Lee E, Eun S, Kim J, Jahng GH, et al. Phantom Acupuncture Induces Placebo Credibility and Vicarious Sensations: a Parallel fMRI Study of Low Back Pain Patients. Scientific reports. 2018;8(1):930.
6. Thomas KJ, MacPherson H, Thorpe L, Brazier J, Fitter M, Campbell MJ, et al. Randomised controlled trial of a short course of traditional acupuncture compared with usual care for persistent non-specific low back pain. BMJ (Clinical research ed). 2006;333(7569):623.
7. Kawase Y, Ishigami T, Nakamura H, Hattori T, Minagawa M, Kouda H. Acupuncture treatment for lower back pain: multi-center randomized controlled trial using sham acupuncture as a control. Journal of the japan society of acupuncture and moxibustion. 2006;56(2):140-9.

- Not about manual acupuncture: 2

1. Macdonald AJ, Macrae KD, Master BR, Rubin AP. Superficial acupuncture in the relief of chronic low back pain. Annals of the Royal College of Surgeons of England. 1983;65(1):44-6.
2. Zaringhalam J, Manaheji H, Rastqar A, Zaringhalam M. Reduction of chronic non-specific low back pain: a randomised controlled clinical trial on acupuncture and baclofen. Chinese medicine. 2010;5:15.

- Not using sham acupuncture or waiting list as controls: 5

© 2023 Lee B et al. *JAMA Network Open.*

1. Luo Y, Yang M, Liu T, Zhong X, Tang W, Guo M, et al. Effect of hand-ear acupuncture on chronic low-back pain: a randomized controlled trial. Journal of traditional Chinese medicine / Chung i tsa chih ying wen pan. 2019;39(4):587-94.
2. Ushinohama A, Cunha BP, Costa LO, Barela AM, Freitas PB. Effect of a single session of ear acupuncture on pain intensity and postural control in individuals with chronic low back pain: a randomized controlled trial. Brazilian journal of physical therapy. 2016;20(4):328-35.
3. Weiß J, Quante S, Xue F, Muche R, Reuss-Borst M. Effectiveness and Acceptance of Acupuncture in Patients with Chronic Low Back Pain: results of a Prospective, Randomized, Controlled Trial. Journal of alternative and complementary medicine (New York, NY). 2013;19(12):935-41.
4. Yun M, Shao Y, Zhang Y, He S, Xiong N, Zhang J, et al. Hegu acupuncture for chronic low-back pain: A randomized controlled trial. Journal of Alternative and Complementary Medicine. 2012;18(2):130-6.
5. Yun M, Xiong N, Guo M, Zhang J, Liu D, Luo Y, et al. Acupuncture at the back-pain-acupoints for chronic low back pain of Peacekeepers in Lebanon: a randomized controlled trial. Journal of musculoskeletal pain. 2012;20(2):107-15.

- Not reporting the outcome of interest in a form suitable for meta-analysis: 3

1. McDonough S, Hunter R, Dhamija S, Walsh D. Manual auricular acupuncture as an adjunct to exercise in people with chronic low back pain: a feasibility study. European journal of pain (London, England). 2009;13:S138-.
2. Thomas K, Thorpe L, MacPherson H. Preliminary findings from a pragmatic randomised controlled trial demonstrate acceptability of acupuncture as a treatment for low back pain. Clin acupunct orient med. 2003;4(1):56.
3. Liu H, Li YP, Hou MJ, Huang WJ, Chen XL, Gao Z, et al. Effect of trigger point acupuncture on pain and functional activity in patients with chronic non-specific low back pain: a randomised controlled trial. Acupunct Med. 2023:9645284221107685.

- Duplicate data: 1

1. Liu Y, Azizi H, Khoursand-Vakilzadeh A, Esmaily H, Bahrami A, Zhao Bai X. A Randomized Controlled Trial of Acupuncture for Chronic Low Back Pain. Journal of alternative and complementary medicine (New York, NY). 2014;20(5):A40-A.

© 2023 Lee B et al. *JAMA Network Open.*

eTable 1. Details of Acupuncture Treatment Method and Funding Source

Study ID	Acupuncture protocol	Treatment points	Depth of insertion	Needle stimulation	Needle retention time	Needle type (number of needle)	Number of treatment session	Frequency	Qualification or experiences on acupuncture	Other interventions in all groups	Study setting	Funding source
Brinkhaus 2006	Semi-standardized	at least 4 bilateral local points (BL20 to 34, BL50 to 54, GB30, GV3 to 6, Huatojiaji, and Shiqizhuixia); and at least 2 bilateral distant points (SI3, BL40, BL60, BL62, KI3, KI7, GB31, GB34, GB41, LV3, GV14, and GV20). If participants were experiencing local or pseudoradicular sensations, at least 2 local points were added. Other acupuncture points, including ear and trigger points, were chosen individually.	NR	De qi, stimulated manually at least once	30 min	not predefined (NR)	12	twice a week for 4 weeks and once a week for next 4 weeks	at least 140 hours of acupuncture training and at least 3 years of clinical experience	take oral NSAIDs, if required	hospital outpatient units in Germany	German social health insurance funds
Cherkin 2009	Semi-standardized	bilateral BL23, BL40, KI3, Ashi point, and GV3	generally between 1-3 cm	De qi, twirling the needles at 10 min and again just prior to needle removal	20 min	0.25 mm X 1.5 inch (8)	10	twice a week for 3 weeks and once a week for next 4 weeks	6 licensed acupuncturists with experience of 4 to 19 years	a self-care book with information on managing flare-ups, exercise, and lifestyle modifications	2 research clinics in western Washington and northern California in USA	NIH Cooperative Agreement (U01 AT 001110)
Cho 2013	Semi-standardized	1) gallbladder meridian pattern: GB12, GB26, GB30, GB34, GB41<br>2) bladder meridian pattern: BL23, BL24, BL25, BL37, BL40<br>3) mixed pattern: ST4, ST36, SP13, SP14, GV3, GV4, GV5, GV24, GV26	5-20 mm, depending on the acupunctu re point	De qi, manual stimulation by bidirectional rotation	15-20 min	0.25 X 40 mm (NR)	12	twice a week for 6 weeks	licensed Korean medicine doctors with at least 3 years of experience	requested to do exercises every day and to try to maintain the correct posture according to the given manual	3 medical hospitals in South Korea	Korea Health Industry Development Institute
Haake 2007	Semi-standardized	fixed points and additional points (from a prescribed list) chosen individually on the basis of traditional	5-40 mm, depending on the site	De qi, manual stimulation	30 min	0.25 X 40 mm or 0.35 X 50 mm (14-20)	10 (5 additional sessions if subjects experienced a 10% to 50%	twice a week for 5 weeks	physicians of various specializations who had at least 140 hours of acupuncture	for acute episodes of pain, only rescue medication was permitted	340 outpatient practices in Germany	German public health insurance companies

© 2023 Lee B et al. *JAMA Network Open.*

		Chinese medicine diagnosis					reduction in pain intensity after the 10th session)		training in Germany			
Itoh 2009	Standardized	BL23, BL25, BL32, BL40, BL60, GB30, GB34	10 mm	De qi, needles inserted using "sparrow pecking" technique until De qi achieved	15 min	0.2 X 40 mm (NR)	5	once a week for 5 weeks	4 years of acupuncture training and 3 to 8 years of clinical experience	None	outpatients at Meiji University of Oriental Medicine Hospital in Japan	NR
Kwon 2007	Individualized	SI3, BL62, BL60, BL40, CV4, BL23, BL52, BL29, CV2, GB30	25-30 mm	De qi, reinforcing and reducing by lifting and thrusting the needle	20 min	0.25 X 40 mm (14)	12	3 times a week for 4 weeks	a Korean medicine doctor	None	Won-Kwang University Hospital in South Korea	Acupuncture, Moxibustion, and Meridian Research Project (K06070) of Korea Institute of Oriental Medicine in 2006
Leibing 2002	Standardized	20 body points – GV3, GV4 (single), BL40, BL60, BL23, BL25, BL32, GB34, SP6, BL31, and Yautungdien (all bilaterally); 6 ear points – os sacrum (38), parasympathic (51), nervus ischiadicus (52), lumbosacrum (54), shenmen (55), and kidney (95) (alternatively on one ear)	10-30 mm (body)	De qi, manually stimulated	30 min (body), 1 week (ear)	0.3 X 40 mm (body), 0.23 mm thickness (ear) (26)	20	5 times a week for 2 weeks and once a week for next 10 weeks	an experienced Taiwanese physician	26 sessions of standardized active physiotherapy at 30 min each over 12 weeks#	at outpatient clinic at Department of Orthopedics, University Goettingen in Germany	Ministry of Education, Science, Research and Technology (BMBFT), Federal Republic of Germany (01 KT 9407)
Molsberger 2002	Semi-standardized	BL23, BL25, GB30, BL40, BL60, GB34; up to 4 Ashi points	1-10 cm	De qi, manually stimulated	30 min	NR (NR)	12	3 times a week for 4 weeks	an experienced medical doctor who had studied acupuncture in China	conventional orthopedic therapy#	from consecutive inpatients of a rehabilitation hospital in Germany	German Ministry of Education, Science and Research
Witt 2006	Individualized	decided by acupuncturist	NR	NR	NR	NR (decided by acupunctur ist)	maximum 15	NR	physicians required to have at least a German diploma representing 140 hours of certified	as needed	NR	a number of German social health insurance funds

© 2023 Lee B et al. *JAMA Network Open.*

									acupuncture education			
Yu 2020	Semi-standardized	GV3, bilateral BL23, BL40, KI3, and 1-3 Ashi points bilaterally	NR	De qi, twirling the needles at 10 min and again just prior to needle removal	25 min	NR (9-13)	6	twice a week for 2 weeks and once a week for next 2 weeks	a licensed acupuncturist in Massachusetts General Hospital	allowed to continue their existing medication	NR	National Institutes of Health (No. P01 AT006663, No. R01 AT008563, No. R33 AT009310, No. R33AT009341, No. R34DA046635, and No. R01AG063975)

*In the original study, the acupuncture therapy group was divided into (semi)standardized and individualized acupuncture therapy groups. However, only data corresponding to the (semi)standardized acupuncture therapy group that meets the question of this study were included in the analysis.

#Although these studies were originally a 3-arm trial, only the acupuncture and sham acupuncture groups were included in the analysis as these treatments provided to all interventions were considered as therapeutic interventions.

Abbreviations. NR, not reported; NSAIDs, non-steroidal anti-inflammatory drugs.

© 2023 Lee B et al. *JAMA Network Open.*

eTable 2. Results of Testing Inconsistency at the Local Level Through the Node Splitting Method

Outcome	Side	Direct		Indirect		Difference		p value
		Coefficient	SE	Coefficient	SE	Coefficient	SE	
Pain	Sham AT(sham) WL	0.53033	0.234379	0.225368	0.189423	0.3049616	0.301275	0.311
Function	Sham AT(sham) WL	0.27071	0.167	0.27869	0.0764343	-0.0079813	0.18365	0.965

Abbreviations. Sham AT(sham), sham acupuncture needling at different points compared with the acupuncture group; WL, waiting list.

© 2023 Lee B et al. *JAMA Network Open.*

eTable 3. League Table for Pairwise Meta-Analysis (Right Upper Part) and Network Meta-Analysis (Left Lower Part)

Estimates: Using the Random-Effects Model: Pain

WL	**-0.67 (-0.92, -0.42)**	-	**-0.54 (-0.87, -0.21)**
**-0.68 (-0.93, -0.43)**	AT	-0.12 (-0.34, 0.10)	**0.35 (0.17, 0.53)**
**-0.80 (-1.26, -0.34)**	-0.12 (-0.51, 0.26)	Sham AT(verum)	-
**-0.34 (-0.63, -0.06)**	**0.33 (0.15, 0.52)**	**0.45 (0.03, 0.88)**	Sham AT(sham)

The results are presented as the standardized mean difference (95% confidence interval). The comparison must be read from left to right. A standardized mean difference greater than zero indicates that treatment on the left is favored in both pairwise and network meta-analyses. The values in bold text indicate statistical significance.

Abbreviations. AT, acupuncture therapy; Sham AT(sham), sham acupuncture needling at different points compared with the acupuncture group; Sham AT(verum), sham acupuncture needling at the same acupuncture points as the acupuncture group; WL, waiting list.

© 2023 Lee B et al. *JAMA Network Open.*

eTable 4. League Table for Pairwise Meta-Analysis (Right Upper Part) and Network Meta-Analysis (Left Lower Part)

Estimates: Using the Fixed-Effects Model: Pain

WL	**-0.58 (-0.65, -0.50)**	-	**-0.54 (-0.87, -0.21)**
**-0.58 (-0.65, -0.50)**	AT	-0.12 (-0.34, 0.10)	**0.26 (0.15, 0.37)**
**-0.70 (-0.94, -0.47)**	-0.12 (-0.34, 0.10)	Sham AT(verum)	-
**-0.33 (-0.46, -0.20)**	**0.25 (0.14, 0.36)**	**0.37 (0.12, 0.62)**	Sham AT(sham)

The results are presented as the standardized mean difference (95% confidence interval). The comparison must be read from left to right. A standardized mean difference greater than zero indicates that treatment on the left is favored in both pairwise and network meta-analyses. The values in bold text indicate statistical significance.

Abbreviations. AT, acupuncture therapy; Sham AT(sham), sham acupuncture needling at different points compared with the acupuncture group; Sham AT(verum), sham acupuncture needling at the same acupuncture points as the acupuncture group; WL, waiting list.

eTable 5. League Table for Pairwise Meta-Analysis (Right Upper Part) and Network Meta-Analysis (Left Lower Part)

Estimates: Using the Random-Effects and Fixed-Effects Models: Function

WL	**-0.41 (-0.49, -0.34)**	**-**	-0.26 (-0.59, 0.07)
**-0.41 (-0.49, -0.34)**	AT	-0.17 (-0.39, 0.05)	**0.14 (0.02, 0.26)**
**-0.58 (-0.82, -0.35)**	-0.17 (-0.39, 0.05)	Sham AT(verum)	**-**
**-0.28 (-0.41, -0.14)**	**0.13 (0.02, 0.25)**	**0.30 (0.05, 0.56)**	Sham AT(sham)

Identical results were obtained for both random-effects and fixed-effects models. The results are presented as the standardized mean difference (95% confidence interval). The comparison must be read from left to right. A standardized mean difference greater than zero indicates that treatment on the left is favored in both pairwise and network meta-analyses. The values in bold text indicate statistical significance.

Abbreviations. AT, acupuncture therapy; Sham AT(sham), sham acupuncture needling at different points compared with the acupuncture group; Sham AT(verum), sham acupuncture needling at the same acupuncture points as the acupuncture group; WL, waiting list.

© 2023 Lee B et al. *JAMA Network Open.*

eTable 6. The Quality of Evidence for the Main Findings: Pain

Comparison		Direct evidence	Indirect evidence	Network meta-analysis
AT	Sham AT(verum)	Moderate<br>Risk of bias (-1)	Moderate<br>Risk of bias (-1)	Low<br>Risk of bias (-1)<br>Imprecision (-1)
AT	Sham AT(sham)	Moderate<br>Risk of bias (-1)	Moderate<br>Risk of bias (-1)	Moderate<br>Risk of bias (-1)
AT	WL	Moderate<br>Risk of bias (-1)	Moderate<br>Risk of bias (-1)	Moderate<br>Risk of bias (-1)
Sham AT(verum)	Sham AT(sham)	-	Moderate<br>Risk of bias (-1)	Moderate<br>Risk of bias (-1)
Sham AT(verum)	WL	-	Moderate<br>Risk of bias (-1)	Moderate<br>Risk of bias (-1)
Sham AT(sham)	WL	Moderate<br>Risk of bias (-1)	Moderate<br>Risk of bias (-1)	Moderate<br>Risk of bias (-1)

Abbreviations. AT, acupuncture therapy; Sham AT(sham), sham acupuncture needling at different points compared with the acupuncture group; Sham AT(verum), sham acupuncture needling at the same acupuncture points as the acupuncture group; WL, waiting list.

© 2023 Lee B et al. *JAMA Network Open.*

eTable 7. The Quality of Evidence for the Main Findings: Function

Comparison		Direct evidence	Indirect evidence	Network meta-analysis
AT	Sham AT(verum)	Moderate<br>Risk of bias (-1)	Moderate<br>Risk of bias (-1)	Low<br>Risk of bias (-1)<br>Imprecision (-1)
AT	Sham AT(sham)	Moderate<br>Risk of bias (-1)	Moderate<br>Risk of bias (-1)	Moderate<br>Risk of bias (-1)
AT	WL	Moderate<br>Risk of bias (-1)	Moderate<br>Risk of bias (-1)	Moderate<br>Risk of bias (-1)
Sham AT(verum)	Sham AT(sham)	-	Moderate<br>Risk of bias (-1)	Moderate<br>Risk of bias (-1)
Sham AT(verum)	WL	-	Moderate<br>Risk of bias (-1)	Moderate<br>Risk of bias (-1)
Sham AT(sham)	WL	Moderate<br>Risk of bias (-1)	Moderate<br>Risk of bias (-1)	Moderate<br>Risk of bias (-1)

Abbreviations. AT, acupuncture therapy; Sham AT(sham), sham acupuncture needling at different points compared with the acupuncture group; Sham AT(verum), sham acupuncture needling at the same acupuncture points as the acupuncture group; WL, waiting list.

© 2023 Lee B et al. *JAMA Network Open.*

eFigure 1. Risk of Bias Summary for All Included Studies

Risk of Bias Tool

	Random sequence generation (selection bias)	Allocation concealment (selection bias)	Blinding of participants (performance bias)	Blinding of personnel (performance bias)	Blinding of outcome assessment (detection bias)	Incomplete outcome data (attrition bias)	Selective reporting (reporting bias)	Other bias
Brinkhaus 2006	+	+	−	+	−	+	+	−
Cherkin 2009	+	+	+	+	+	+	+	+
Cho 2013	+	+	+	+	+	−	+	−
Haake 2007	+	+	+	+	+	+	+	+
Itoh 2009	+	?	−	+	−	−	+	+
Kwon 2007	+	?	+	+	+	+	+	+
Leibing 2002	+	?	+	+	+	+	+	+
Molsberger 2002	+	+	+	+	+	+	+	+
Witt 2006	+	+	−	+	−	+	+	+
Yu 2020	+	+	+	+	+	−	+	+

Low, unclear, and high risk, respectively, are represented with the following symbols: “+”, “?”, and “-”.

© 2023 Lee B et al. *JAMA Network Open.*

- Revised Risk Of Bias Tool (RoB 2)

	Bias arising from the randomization process	Bias due to deviations from intended interventions (effect of assignment to intervention)	Bias due to missing outcome data	Bias in measurement of the outcome	Bias in selection of the reported result	Overall bias
Brinkhaus 2006	+	?	+	−	+	−
Cherkin 2009	+	?	+	+	+	?
Cho 2013	?	?	+	+	+	?
Haake 2007	+	?	+	+	+	?
Itoh 2009	?	?	?	−	?	−
Kwon 2007	?	?	+	+	?	?
Leibing 2002	?	?	+	+	?	?
Molsberger 2002	+	?	+	+	?	?
Witt 2006	+	?	+	−	?	−
Yu 2020	+	?	+	+	+	?

Low risk, some concerns, and high risk, respectively, are represented with the following symbols: "+", "?", and "-".

© 2023 Lee B et al. *JAMA Network Open.*

eFigure 2. Funnel Plot: Pain

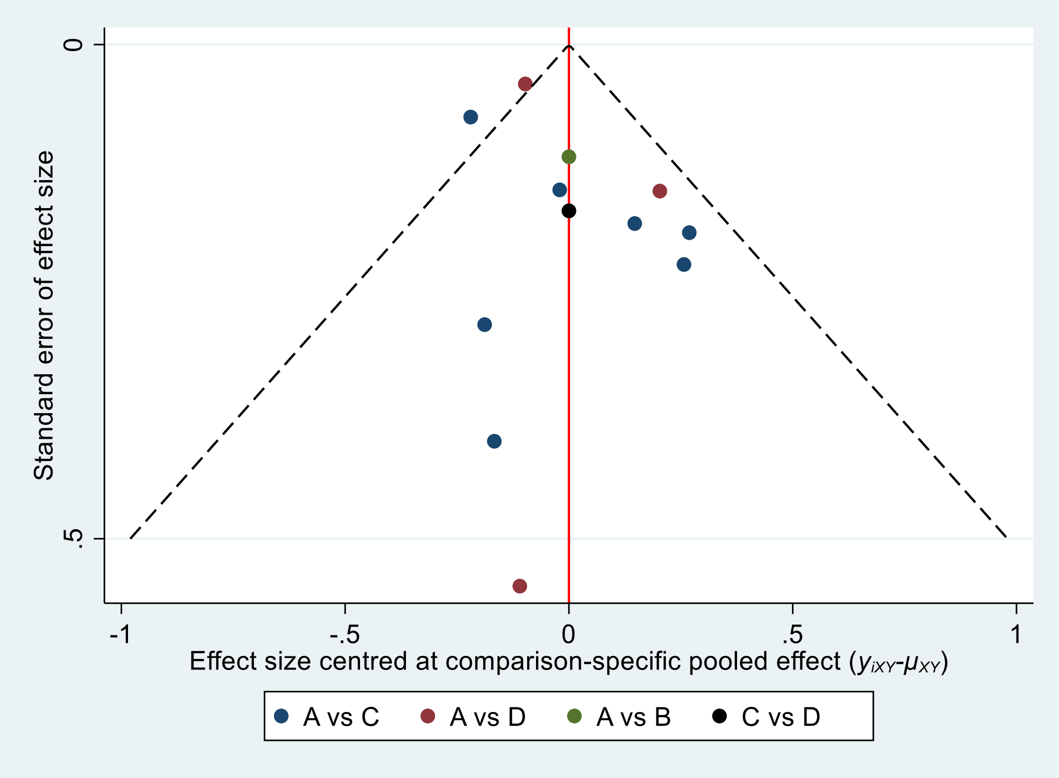

A, acupuncture therapy; B, sham acupuncture needling at the same acupuncture points as the acupuncture group; C, sham acupuncture needling at different points compared with the acupuncture group; D, waiting list.

© 2023 Lee B et al. *JAMA Network Open.*

eFigure 3. SUCRA Plots

(A) Pain

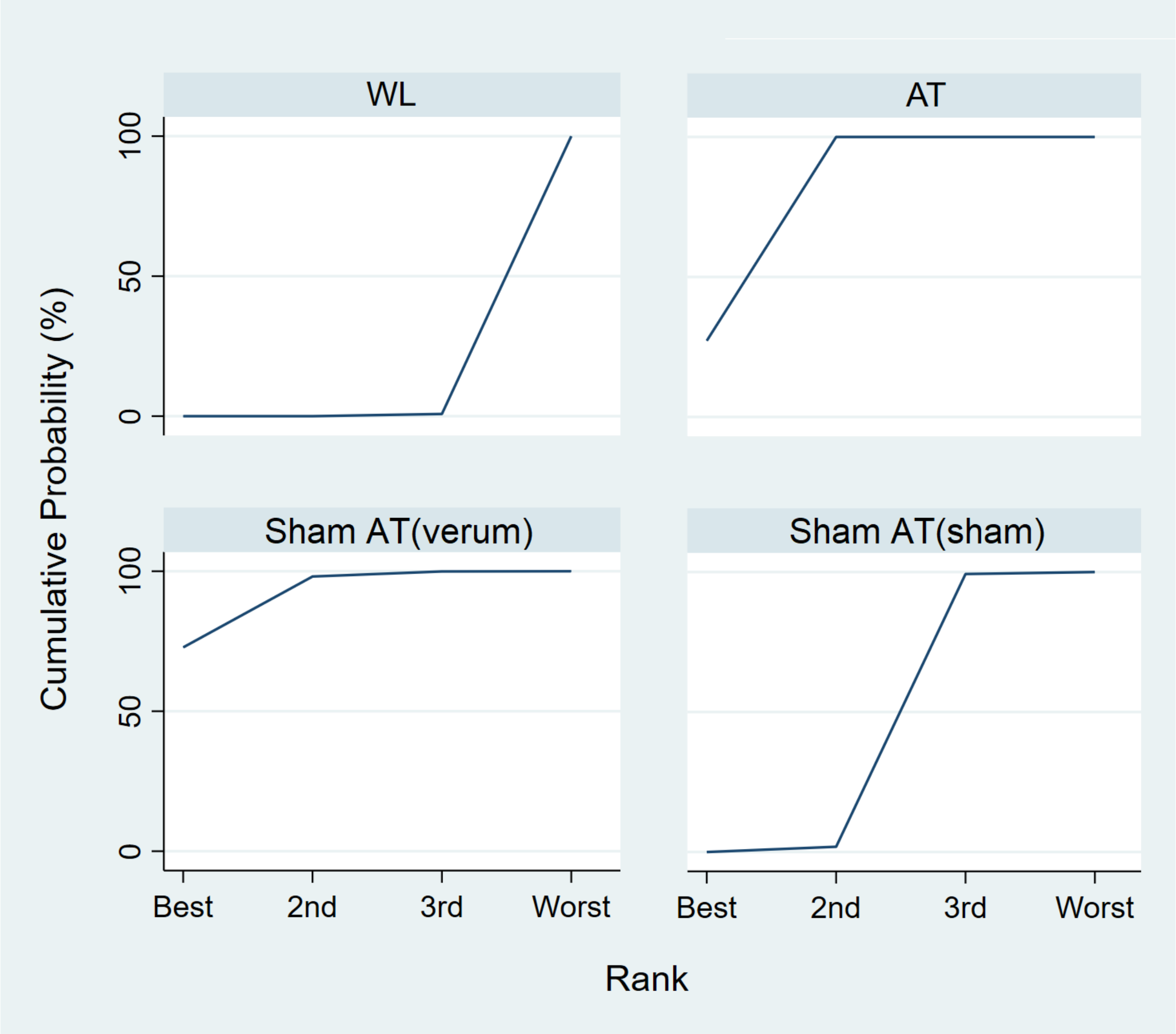

© 2023 Lee B et al. *JAMA Network Open.*

(B) Function

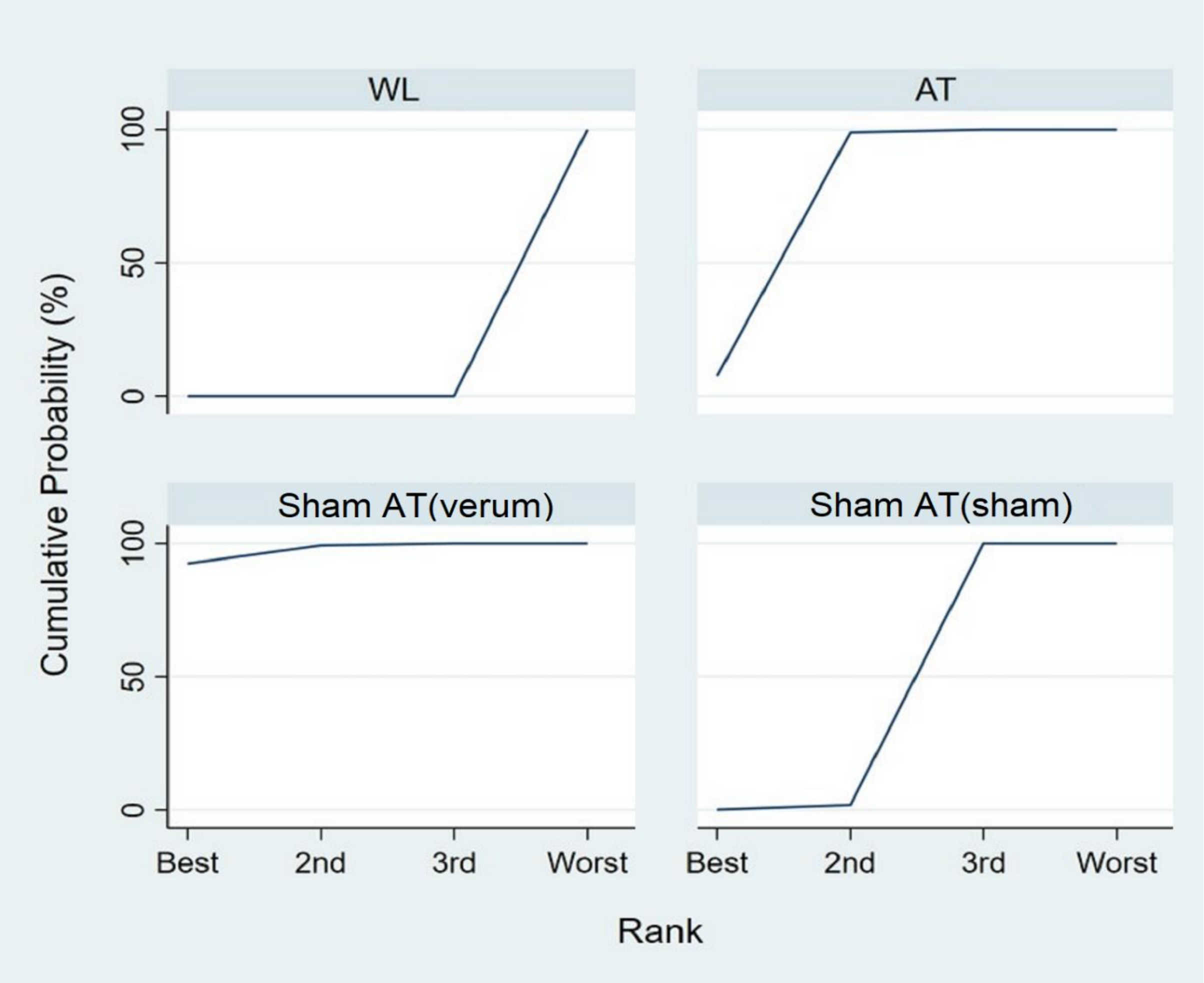

Abbreviations. AT, acupuncture therapy; Sham AT(sham), sham acupuncture needling at different points compared with the acupuncture group; Sham AT(verum), sham acupuncture needling at the same acupuncture points as the acupuncture group; WL, waiting list.

*SUCRA is a numeric presentation of the overall ranking and presents a single number associated with each treatment. SUCRA values range from 0 to 100%. The higher the SUCRA value, and the closer to 100%, the higher the likelihood that a therapy is in the top rank or one of the top ranks; the closer to 0 the SUCRA value, the more likely that a therapy is in the bottom rank, or one of the bottom ranks.

© 2023 Lee B et al. *JAMA Network Open.*

eFigure 4. Interval Plots After Sensitivity Analysis Excluding a Pilot Study

(A) Pain

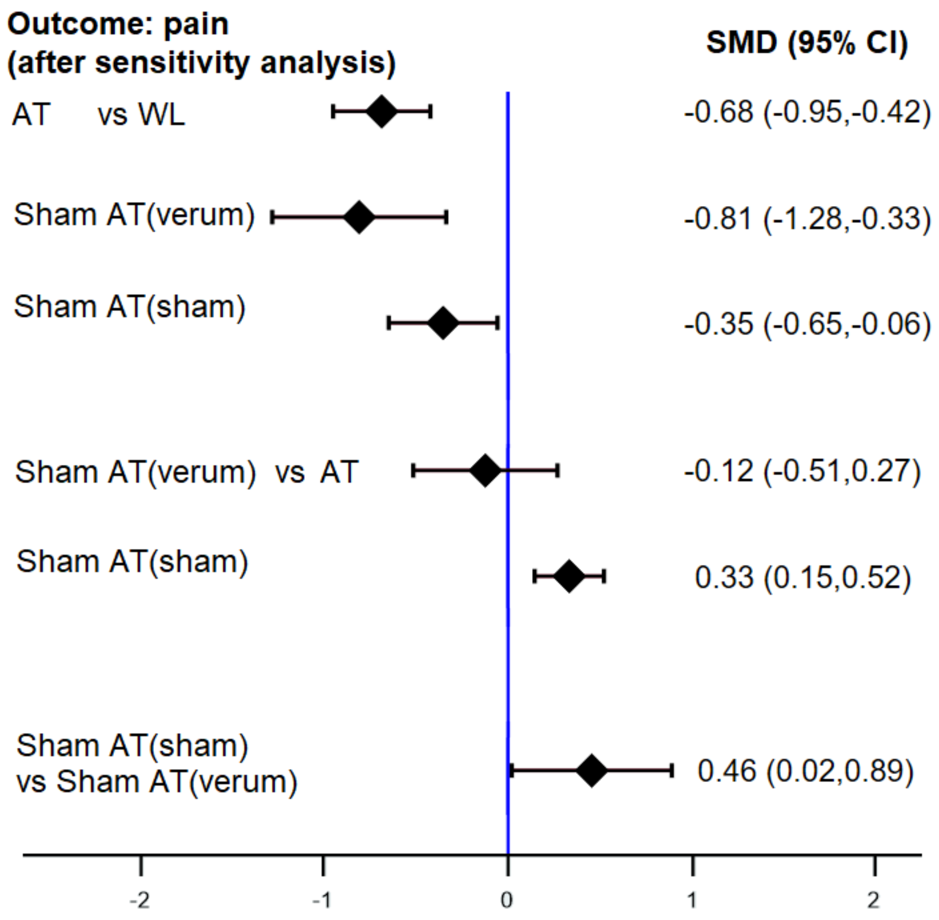

© 2023 Lee B et al. *JAMA Network Open.*

(B) Function

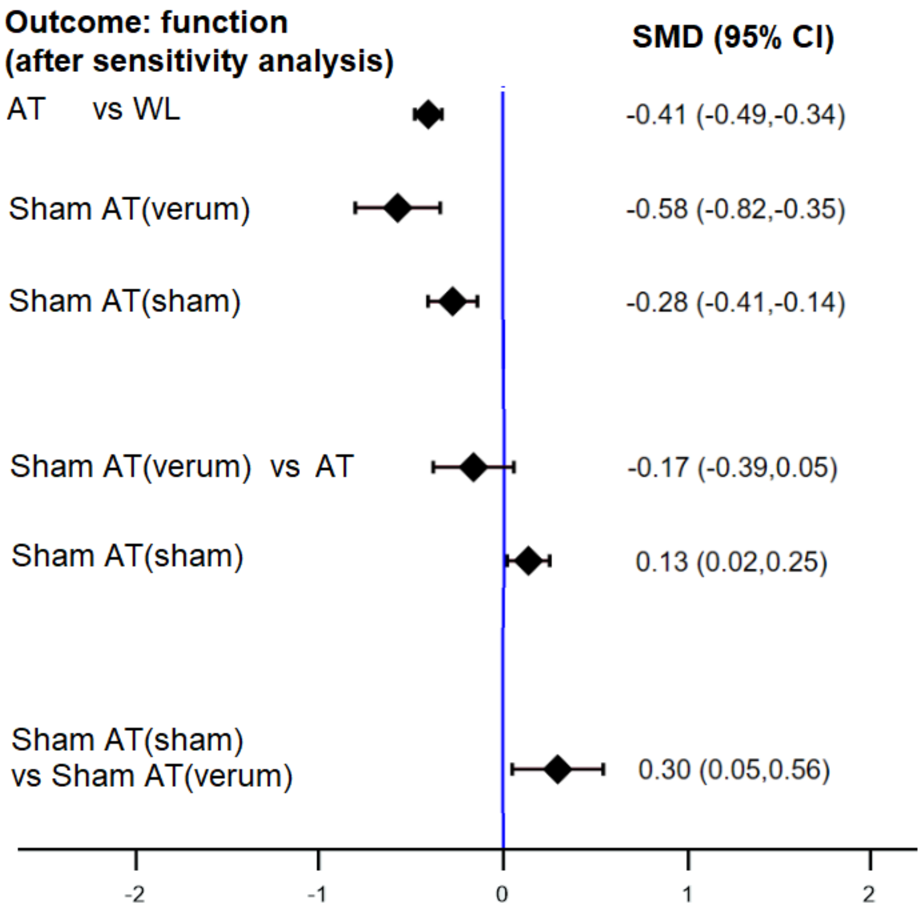

Abbreviations. AT, acupuncture therapy; CI, confidence interval; Sham AT(sham), sham acupuncture needling at different points compared with the acupuncture group; Sham AT(verum), sham acupuncture needling at the same acupuncture points as the acupuncture group; SMD, standardized mean difference; WL, waiting list.

© 2023 Lee B et al. *JAMA Network Open.*
