# Supplementary material for: Needling Point Location Used in Sham Acupuncture for Chronic Nonspecific Low Back Pain: A Systematic Review and Network Meta-Analysis
Source: JAMA Netw Open. 2023 Sep 6;6(9):e2332452. doi: 10.1001/jamanetworkopen.2023.32452 (PMC10483312; doi:10.1001/jamanetworkopen.2023.32452)
Supplement: Supplement 2. — Data Sharing Statement [file jamanetwopen-e2332452-s002.pdf]

Data Sharing Statement

Lee. Needling Point Location Used in Sham Acupuncture for Chronic Nonspecific Low Back Pain. *JAMA Netw Open*. Published September 06, 2023.
doi:10.1001/jamanetworkopen.2023.32452

Data
Data available: Yes
Data types: Data (not involving human participants)
How to access data: The authors confirm that the data supporting the findings of this study are available within the article and its supplementary materials.
When available: With publication

Supporting Documents
Document types: None

Additional Information
Who can access the data: anyone
Types of analyses: for any purpose
Mechanisms of data availability: without investigator support
